# Pain control in trauma patients in emergency departments: current status and influencing factors

**DOI:** 10.3389/fpubh.2025.1641198

**Published:** 2025-10-20

**Authors:** Zinian Wei

**Affiliations:** Emergency Department, Suzhou Research Center of Medical School, Suzhou Hospital, Affiliated Hospital of Medical School, Nanjing University, Suzhou, China

**Keywords:** trauma patients, emergency department, pain control, influencing factors, nurses’ attitudes

## Abstract

**Objective:**

To investigate the current status of pain control in trauma patients in the emergency department, as well as nurses’ attitudes, behaviors, and influencing factors regarding pain management, with the aim of improving the quality of emergency care.

**Methods:**

A single-center cross-sectional study was conducted. Using convenience sampling, 245 trauma patients admitted to the emergency department of Suzhou Research Center of Medical School, Suzhou Hospital, Affiliated Hospital of Medical School, between January and December 2024 were enrolled, along with 79 emergency nurses. Patients were assessed using the Modified Early Warning Score (MEWS) and the Facial Rating Scale (FRS) for pain. Questionnaires were administered to both patients and nurses. Statistical analyses included descriptive statistics, t-tests, logistic regression, and multiple linear regression.

**Results:**

The majority of patients were male (68.16%) and aged 18–60 years (80.82%). The most common injuries were limb (41.63%) and chest-abdominal (31.43%), with traffic accidents as the leading cause (40.00%). Analgesic administration rates increased with MEWS scores (0% in MEWS 0–2, 44.0% in 5–6, and 67.9% in ≥9). However, patient satisfaction did not increase correspondingly (29.21% in MEWS 3–4, 34.00% in 5–6). Nurses expressed strong concern that analgesia may mask clinical conditions (mean score 4.29 ± 0.56). Logistic regression showed that main injury site (OR = 0.69, *p* = 0.014), injury type (OR = 2.18, *p* = 0.001), analgesia request (OR = 1.68, *p* = 0.004), and injury manifestation (OR = 1.62, *p* = 0.003) were independent predictors of satisfaction. Multiple linear regression confirmed analgesia request (*β* = 0.32, *p* < 0.001) and obvious injury manifestation (β = 0.25, *p* = 0.002) as positive predictors, while limb injuries predicted lower satisfaction (*β* = −0.19, *p* = 0.008).

**Conclusion:**

Pain control in emergency trauma patients is influenced by injury severity, nurses’ attitudes, and patient-related factors. Comprehensive pain assessment, nurse training, and consideration of patient requests and injury characteristics are essential to improving emergency pain management.

## 1 Introduction

Trauma, resulting from mechanical injury factors, leads to the disruption of tissue structure integrity or functional impairment in the body (1). Up to 91% of trauma patients experience varying degrees of pain upon arrival at the emergency department (2). This pain significantly impacts patients’ physiological and psychological states (3, 4). For example, it can trigger overexcitement of the adrenergic system, leading to a series of pathophysiological reactions and even fainting in severe cases, thereby exacerbating injury and its complexity. Thus, pain control is crucial for the treatment and rehabilitation of trauma patients.

The emergency department is often the first stop for trauma patients in the hospital. However, studies indicate that the pain of emergency trauma patients remains inadequately managed (3). Notably, 86% of trauma patients who enter the emergency department with pain still experience pain when they leave, and there is a notable shortage in the use of analgesic medications for severely traumatized patients (4). In the emergency department, general trauma patients usually receive treatment in the emergency clinic or treatment room and are then transferred to other departments or discharged. Severe trauma patients, on the other hand, are admitted to the emergency room for close observation; emergency treatments such as hemostasis, dressing, fixation, and fluid replacement; and await transfer. They typically stay in the emergency room for several hours. Nurses, as the medical staff in closest contact with these patients, play a vital role in pain control. Regrettably, research shows that the lack of effective pain control in emergency patients is associated with several factors, including medical staff underestimating pain, lacking pain-related knowledge, holding a passive attitude toward analgesia, the tight schedule in the emergency department, and the absence of proper analgesia policies (5). Furthermore, multiple studies have revealed that emergency nurses demonstrate a lower level of pain-related cognition and practice compared to their counterparts in other departments (6).

In the high-paced emergency rescue process, particularly within the emergency rescue room setting, pain control in trauma patients often fails to receive sufficient attention, resulting in pain being underestimated (7). Patients thus endure excruciating pain beyond their tolerance, which may cause severe adverse psychological and physiological reactions and reduce their satisfaction with emergency medical services (8). However, there remains a distinct lack of studies specifically investigating the current status and influencing factors of pain management within the unique context of the emergency rescue room.

Against this backdrop, this study aims to investigate the current status of pain control in trauma patients in the emergency room, as well as nurses’ attitudes, behaviors, and influencing factors regarding pain control. The findings of this research are expected to draw the attention of medical staff to the pain of such patients and provide a reference for enhancing nurses’ subjective initiative in pain control, ultimately improving the quality of emergency care for trauma patients.

## 2 Methods

### 2.1 Study design and participants

This cross-sectional study was conducted from January to December 2024 at the emergency room of Suzhou Research Center of Medical School, Suzhou Hospital, and Affiliated Hospital of Medical School. A non-probability convenience sampling method was utilized to recruit participants from the emergency room over a 12-month period.

The sample size was calculated *a priori* using G*Power software (version 3.1.9.7) for two independent study groups. Based on an effect size of 0.3 (medium), *α* error probability of 0.05, and statistical power of 0.8, the analysis indicated minimum requirements of 200 patients and 70 nurses. To account for potential non-responses and missing data, we increased the sample size by approximately 20%, resulting in the final recruitment of 245 trauma patients and 79 nurses (9).

Patient inclusion criteria comprised: trauma patients seeking emergency treatment during the study period, consciousness and ability to comprehend and respond to survey questions, and age 18 years or older. Exclusion criteria included: comatose patients, those with severe cognitive impairments (e.g., advanced dementia or severe intellectual disabilities), and patients with language barriers that could not be resolved via interpretation services. For nurses, inclusion criteria were: working in the emergency department of a Class-A tertiary hospital for at least 1 year and current employment in the emergency department during the study period. Exclusion criteria were: nurses on long-term leave (e.g., maternity, sick, or sabbatical leave) and those who had been transferred to the emergency department less than 1 year prior (10).

### 2.2 Data collection instruments

The Modified Early Warning Score (MEWS) was used at admission to objectively quantify and stratify the overall clinical severity and physiological instability of trauma patients. This assessment provides a standardized, composite measure of a patient’s acute condition, which is crucial for guiding initial triage decisions, allocating appropriate levels of monitoring and care, and serving as a foundational objective variable for analyzing the provision and effectiveness of pain control interventions across different injury severity levels. The MEWS evaluates parameters including heart rate, systolic blood pressure, respiratory rate, body temperature, and level of consciousness. Each parameter is scored from 0 to 3, with a total score ranging from 0 to 15; higher scores indicate greater physiological derangement and clinical severity (11).

The Facial Expression Rating Scale (FRS) was used to measure patients’ pain levels, with scores ranging from 0 (no pain) to 5 (severe pain) (12). This tool was chosen because it provides a simple, intuitive, and rapid assessment method, particularly suitable for trauma patients who may have difficulty providing detailed verbal or written responses due to pain, distress, or language barriers. Although more commonly used tools such as the Numeric Rating Scale (NRS) and Visual Analog Scale (VAS) were considered, the FRS was deemed more appropriate in this high-acuity setting for its clinical feasibility and ease of use across diverse patient populations, including those with limited health literacy or cognitive overload (13, 14).

Satisfaction with pain control was evaluated using a 5-point Likert scale (1 = very dissatisfied to 5 = very satisfied).

A structured data collection form was specifically designed to document analgesic treatments received by patients during their stay in the emergency room. This form captured detailed information including: the class and name of analgesic medication administered (e.g., opioids, non-steroidal anti-inflammatory drugs), dosage, route of administration (e.g., intravenous, oral), time of administration relative to admission, and any non-pharmacological pain interventions utilized. This data was meticulously verified against electronic medical records and medication administration charts by trained research staff to ensure accuracy and completeness.

The questionnaires for both patients and nurses were developed based on a comprehensive literature review and consultations with experienced emergency department nurses. The nurse attitude scale consisted of 14 items rated on a 5-point scale to assess concerns such as analgesia masking the condition and pain not being a priority (15). Content validity was established through expert review, and internal consistency reliability (Cronbach’s *α*) was 0.82 for the patient questionnaire and 0.79 for the nurse questionnaire.

### 2.3 Procedures

Participants were accessed during their stay in the emergency room. Patients were approached upon stabilization, and nurses were invited during their shifts. To prevent information bias and ensure data quality, several stringent measures were implemented throughout the data collection process. Firstly, all research assistants received standardized training on the objective administration of the questionnaires and the consistent application of the MEWS assessment criteria. Secondly, data collection was structured to minimize variability: patients were assessed at a consistent time point (before discharge from the emergency room), while nurses completed their questionnaires during breaks or after shifts to avoid disruption of clinical duties. Questionnaires were administered in a quiet and private area within the emergency department to ensure confidentiality and minimize social desirability bias. Furthermore, anonymous administration was maintained by using coded identifiers, and completed forms were collected in sealed boxes to protect participant anonymity. To maintain independent assessment and minimize potential bias, nurses were not blinded to the patients’ overall clinical condition (as this is integral to their clinical role) but they did not have access to the specific responses provided by patients on their self-assessment questionnaires during the study period. Finally, the inclusion of objective physiological parameters from the MEWS assessment served to complement and validate subjective reports. The head nurses assisted in coordinating the distribution, and all questionnaires were collected within 2 weeks of distribution.

### 2.4 Ethical considerations

The study protocol was approved by the Ethics Committee of Suzhou Research Center of Medical School, Suzhou Hospital, Affiliated Hospital of Medical School (Approval No. IRB2022134; Date of Approval: August 20, 2022). All participants provided written informed consent after receiving detailed information about the study purpose, procedures, and their rights. Confidentiality and anonymity were ensured by using coded identifiers instead of personal information. Participation was voluntary, and participants could withdraw at any time without affecting their medical care or employment.

### 2.5 Data analysis

Data were analyzed using SPSS version 27.0. Descriptive statistics including means, standard deviations, medians, frequencies, and composition ratios were used to summarize the data. A paired t-test was applied to compare patients’ actual pain levels upon leaving the emergency department with their expected pain control levels. An independent-samples t-test was used to compare differences in pain control-related perceptions between patients and nurses. These tests were selected for their appropriateness in comparing means between related and independent groups, respectively. To identify factors independently associated with satisfaction with pain control, multiple logistic regression analysis was performed with satisfaction status (satisfied vs. dissatisfied) as the dichotomous dependent variable. Additionally, a multiple linear regression analysis was conducted with the continuous satisfaction score (range 1–5) as the dependent variable to provide a more nuanced understanding of the relationships between variables and the degree of satisfaction. For both models, independent variables included in the initial models were those showing significant associations (*p* < 0.05) in univariate analyses. A forced entry method was used for all significant predictors. The models’ goodness-of-fit was assessed using the Hosmer-Lemeshow test for logistic regression and ANOVA for linear regression. Multicollinearity among independent variables was checked using variance inflation factors (VIF), with a VIF < 5 considered acceptable. The assumptions of linearity of logit for continuous variables and the absence of influential outliers were examined using residual analysis and leverage plots. For the linear regression, normality of residuals was confirmed using the Shapiro–Wilk test. Results are presented as adjusted odds ratios (ORs) with corresponding 95% confidence intervals (CIs) for the logistic regression, and as unstandardized/standardized coefficients with *p*-values for the linear regression. The overall model performance was evaluated using Nagelkerke’s R^2^ for logistic regression and adjusted R^2^ for linear regression, while discriminative ability of the logistic model was assessed by the area under the receiver operating characteristic curve (AUC).

## 3 Results

### 3.1 Basic information of trauma patients in the emergency room

A total of 245 trauma patients were included in this study. The basic demographic and clinical characteristics of the participants are summarized in Table 1. The cohort was predominantly male (68.16%) and mostly aged between 18 and 60 years (80.82%). The median length of stay in the emergency room was 6.12 h (IQR: 4.25–8.70). Regarding educational background, patients with junior high school education constituted the largest proportion (35.51%). In terms of occupation, farmers (29.39%) and workers (25.71%) were the most represented groups. Limb injuries (41.63%) and chest/abdominal injuries (31.43%) were the most common injury sites, with contusions (44.08%) being the predominant injury type. Traffic accidents (40.00%) and work-related injuries (29.80%) were the leading causes of trauma.

**Table 1:** Basic characteristics of trauma patients (*n* = 245).

Characteristic	Category	*n* (%)/Median (IQR)
Demographics		
Gender	Male	167 (68.16)
	Female	78 (31.84)
Age (years)	<18	29 (11.84)
	18–60	198 (80.82)
	≥60	18 (7.35)
Marital status	Married	157 (64.08)
	Unmarried	82 (33.47)
	Widowed	6 (2.45)
Education	Primary school or below	42 (17.14)
	Junior high school	87 (35.51)
	High school/Secondary vocational	72 (29.39)
	Junior college or above	44 (17.96)
Occupation	Farmer	72 (29.39)
	Worker	63 (25.71)
	Student	39 (15.91)
	Unemployed	16 (6.53)
	Cadre	11 (4.49)
	Other	44 (17.96)
Clinical features		
Length of stay (hours)	Median (IQR)	6.12 (4.25–8.70)
Injury site	Limb	102 (41.63)
	Chest and abdomen	77 (31.43)
	Head and brain	62 (25.31)
	Spine	47 (19.18)
	Pelvis	26 (10.61)
	Neck	13 (5.30)
Injury type	Contusion	108 (44.08)
	Cutting	46 (18.78)
	Crush	41 (16.73)
	Fall	31 (12.65)
	Laceration	16 (6.53)
	Other	3 (1.22)
Injury cause	Traffic accident	98 (40.00)
	Work-related injury	73 (29.80)
	Fight	22 (9.02)
	Fall	11 (4.49)
	Persecution by others	6 (2.45)
	Natural disaster	4 (1.63)
	Suicide	1 (0.41)

### 3.2 General information of emergency nurses

A total of 79 emergency nurses participated in this study. Their demographic and professional characteristics are presented in Table 2. The participants had a mean age of 27.34 ± 5.32 years, with a median nursing experience of 4.5 years and emergency department experience of 4.0 years. The majority held the title of nurse (63.9%) and had junior college education (71.4%).

**Table 2:** Characteristics of emergency nurses (*n* = 79).

Characteristic	Category	*n* (%)/value
Demographics		
Age (years)	Mean ± SD	27.34 ± 5.32
	Median	26.0
Professional experience		
Nursing experience (years)	Median	4.5
Emergency department experience (years)	Median	4.0
Professional title	Nurse	50 (63.9)
	Nurse practitioner	23 (28.6)
	Head nurse	6 (7.6)
Education	Secondary vocational	9 (10.9)
	Junior college	56 (71.4)
	Undergraduate	14 (17.6)

### 3.3 Analysis of pain control based on MEWS stratification

The analysis of pain control in patients stratified by the MEWS revealed several key findings, with overall comparisons showing significant variations in both analgesic treatment rates (*χ*^2^ = 85.32, *p* < 0.001) and satisfaction levels (*χ*^2^ = 32.15, *p* < 0.001) across MEWS groups. For clinical interpretation, satisfaction was defined as a score of ≥4 on the 5-point Likert scale (very satisfied or satisfied).

For MEWS scores of 0–2 points, representing physiologically stable patients, few received analgesia (0%), yet satisfaction was high (79.63%), with no significant difference between nurse-expected and patient-reported pain levels (*t* = −0.893, *p* = 0.376), suggesting adequate pain alignment in minimal injury cases. In the 3–4 point group (moderate severity), both analgesic treatment (4.49%) and satisfaction rates (29.21%) were notably low, accompanied by a significant pain-perception discrepancy (*t* = −3.258, *p* = 0.002), indicating potential undertreatment and misalignment of pain assessment. The 5–6 point group demonstrated a substantial increase in analgesia rate (44.00%) but persistently low satisfaction (34.00%), with a large, statistically significant pain-perception gap (*t* = −4.285, *p* < 0.001), reflecting ongoing challenges in pain management for this severity tier. In the 7–8 point group, both analgesia (45.83%) and satisfaction rates (70.83%) were moderate, with no significant difference between nurse and patient pain assessments (*t* = 0.714, *p* = 0.482), suggesting improved pain alignment in more critically ill patients. For MEWS ≥ 9 points (most severe), the high analgesia rate (67.86%) coexisted with relatively low satisfaction (42.86%) and a significant reverse pain-perception gap (*t* = 2.548, *p* = 0.017), where nurses anticipated higher pain levels than patients reported, potentially indicating effective analgesia but unmet expectations or other factors affecting satisfaction (Table 3).

**Table 3:** Pain control situation of patients stratified by MEWS.

MEWS score	*n*	Analgesic treatment *n* (%)	Satisfaction rate* *n* (%)	Nurse’s expectation (Mean ± SD)	Patient’s reality (Mean ± SD)	t-value	*p*-value
0–2 points	54	0 (0)	43 (79.63)	1.26 ± 0.12	1.39 ± 0.25	−0.893	0.376
3–4 points	89	4 (4.49)	26 (29.21)	2.55 ± 0.26	3.54 ± 0.52	−3.258	0.002
5–6 points	50	22 (44.00)	17 (34.00)	3.02 ± 0.34	4.28 ± 0.64	−4.285	<0.001
7–8 points	24	11 (45.83)	17 (70.83)	3.54 ± 0.41	3.48 ± 0.41	0.714	0.482
≥9 points	28	19 (67.86)	12 (42.86)	4.02 ± 0.42	3.65 ± 0.56	2.548	0.017

Prior to conducting t-tests, the assumptions of normality and homogeneity of variances were verified using Shapiro–Wilk tests and Levene’s tests, respectively; all data met these parametric assumptions. *Satisfaction defined as score ≥4 on 5-point Likert scale.

### 3.4 Nurses’ attitudes toward emergency pain control

The attitudes of emergency nurses toward pain control are summarized in Table 4. Nurses expressed strong concerns that analgesia might mask patients’ clinical conditions (4.29 ± 0.56) and demonstrated a relatively high belief that pain would not cause additional adverse effects (3.91 ± 0.43). The perception of pain as a non-top-priority issue received a moderate score (3.13 ± 0.47), while skepticism about patients’ pain reports was relatively low (2.17 ± 0.37). Nurses generally rejected the notion that trauma patients do not need pain control (1.43 ± 0.26).

**Table 4:** Nurses’ attitudes toward emergency pain control (*n* = 79).

Attitude statement	Score (Mean ± SD)*
Analgesia may mask the clinical condition	4.29 ± 0.56
Pain will not cause additional adverse effects	3.91 ± 0.43
Pain is not a top-priority issue in emergency care	3.13 ± 0.47
Pain is an inherent element of trauma	2.07 ± 0.53
Patients’ pain manifestations or complaints may be exaggerated or untrue	2.17 ± 0.37
Trauma patients do not need pain control	1.43 ± 0.26

*Scored on a 5-point scale where 1 = strongly disagree, 5 = strongly agree.

### 3.5 Factors influencing emergency pain control satisfaction

Factors associated with satisfaction with pain control management are presented in Table 5. Chi-square analysis revealed several significant factors influencing satisfaction levels. Patients over 60 years showed higher satisfaction rates compared to younger patients (48.81% vs. 41.61%, *p* = 0.013). Female patients reported higher satisfaction than males (51.43% vs. 41.71%, *p* = 0.002). Significant variations were observed across injury sites, with head injury patients demonstrating the highest satisfaction (66.10%) and limb injury patients the lowest (24.39%, *p* < 0.001). Patients with multiple trauma showed significantly higher satisfaction than those with single trauma (66.33% vs. 29.25%, *p* = 0.001). Those who requested analgesia and those with obvious injury manifestations also reported higher satisfaction rates (51.43% vs. 39.29%, *p* = 0.002, and 51.59% vs. 30.68%, *p* = 0.001, respectively).

**Table 5:** Factors associated with satisfaction of pain control (*n* = 245).

Factor	Category	Satisfied *n* (%)	Dissatisfied n (%)	*χ* ^2^	*p*-value
Age (years)				6.22	0.013
	≤60	67 (41.61)	94 (58.39)		
	>60	41 (48.81)	43 (51.19)		
Gender				9.57	0.002
	Male	73 (41.71)	102 (58.29)		
	Female	36 (51.43)	34 (48.57)		
Main injury site				16.70	<0.001
	Head	39 (66.10)	20 (33.90)		
	Spine	21 (56.76)	16 (43.24)		
	Chest and Back	16 (30.19)	37 (69.81)		
	Waist and Abdomen	22 (40.00)	33 (60.00)		
	Limb	10 (24.39)	31 (75.61)		
Injury type				11.88	0.001
	Multiple trauma	65 (66.33)	33 (33.67)		
	Single trauma	43 (29.25)	104 (70.75)		
Analgesia request				9.28	0.002
	Yes	54 (51.43)	51 (48.57)		
	No	55 (39.29)	85 (60.71)		
Injury manifestation				11.32	0.001
	Obvious	81 (51.59)	76 (48.41)		
	Hidden	27 (30.68)	61 (69.32)		

### 3.6 Multiple regression analysis results

A binary logistic regression analysis was performed to identify factors independently associated with satisfaction of pain control (dependent variable: satisfied vs. dissatisfied). The model demonstrated good predictive performance, with a Nagelkerke R^2^ value of 0.28, indicating that the included variables explained approximately 28% of the variance in pain control satisfaction. The Hosmer-Lemeshow test yielded a non-significant result (*χ*^2^ = 7.24, *p* = 0.51), confirming good model fit. The AUC was 0.81 (95% CI, 0.75–0.87), demonstrating excellent discriminative ability (Table 6).

**Table 6:** Results of multiple regression analysis.

Variable	Regression coefficient	Standard error	Wald χ^2^	*p*-value	OR	95% CI
Gender	0.25	0.22	1.32	0.251	1.28	0.84–1.95
Main injury site	−0.37	0.15	6.05	0.014	0.69	0.51–0.93
Injury type	0.78	0.24	10.42	0.001	2.18	1.36–3.50
Analgesia request	0.52	0.18	8.36	0.004	1.68	1.18–2.39
Injury manifestation	0.48	0.16	9.00	0.003	1.62	1.18–2.22

Model fit statistics: Nagelkerke *R*^2^ = 0.28; Hosmer-Lemeshow test: *χ*^2^ = 7.24, *p* = 0.51; AUC = 0.81 (95% CI, 0.75–0.87).

According to the multiple regression analysis, sex had no significant effect (*p* = 0.251). The main injury site was a key factor. With a regression coefficient of −0.37 (negative correlation), the odds ratio (OR) was 0.69. Each unit change in this variable decreased the odds of the outcome, and the 95% confidence interval (0.51–0.93), which did not include 1, confirmed its significance. The injury type was significant (*p* = 0.001). The regression coefficient of 0.78 and an OR of 2.18 indicated that patients with multiple traumatic events were more likely to experience the outcome than those with a single traumatic event, with a 95% CI of 1.36–3.50. Patients who requested analgesia were 1.68 times more likely to have a certain outcome (*p* = 0.004), with a 95% CI of 1.18–2.39. Injury manifestation also significantly affects the outcome. A regression coefficient of 0.48 and *p* = 0.003 indicated that patients with obvious injury manifestations were 1.62 times more likely to have the outcome, with a 95% CI of 1.18–2.22.

### 3.7 Multivariate linear regression analysis of factors influencing satisfaction scores

To further elucidate the complex relationships between patient factors, clinical management, and the degree of satisfaction with pain control, a multiple linear regression was performed with the continuous satisfaction score (range: 1–5) as the dependent variable. The model included the same predictor variables as the logistic regression model for consistency. The overall regression was statistically significant (*F*(5, 239) = 8.92, *p* < 0.001), with an adjusted R^2^ of 0.22, indicating that the model explains approximately 22% of the variance in satisfaction scores.

As shown in Table 7, three variables emerged as significant independent predictors of higher satisfaction scores. Requesting analgesia was associated with the largest increase in satisfaction (*β* = 0.32, *p* < 0.001). The presence of obvious injury manifestations was also a significant positive predictor (*β* = 0.25, *p* = 0.002). Conversely, limb injuries were significantly associated with lower satisfaction scores compared to other injury sites (*β* = −0.19, *p* = 0.008). Injury type (multiple vs. single) and patient gender did not reach statistical significance in this continuous analysis.

**Table 7:** Results of multiple linear regression analysis for predictors of satisfaction score.

Predictor variable	Unstandardized B	Standard error	Standardized β	t-value	*p*-value	95% CI for B
(Constant)	2.15	0.28	-	7.68	<0.001	1.60 to 2.70
Analgesia request (Yes)	0.68	0.16	0.32	4.25	<0.001	0.36 to 1.00
Injury manifestation (Obvious)	0.52	0.17	0.25	3.06	0.002	0.18 to 0.86
Main injury site (Limb)	−0.41	0.15	−0.19	−2.73	0.008	−0.71 to −0.11
Injury type (Multiple)	0.22	0.14	0.11	1.57	0.118	−0.06 to 0.50
Gender (Female)	0.19	0.13	0.09	1.46	0.146	−0.07 to 0.45

Model summary: *R*^2^ = 0.24, Adjusted R^2^ = 0.22, SE of estimate = 0.87. Model statistics: F (5, 239) = 8.92, *p* < 0.001.

## 4 Discussion

This study identified several critical patterns in emergency trauma pain management, revealing that while analgesic treatment rates generally increased with physiological severity as measured by MEWS, this did not consistently translate to higher patient satisfaction. The most significant pain-assessment discrepancies occurred in patients with moderate physiological compromise (MEWS 3–6), where nurses substantially underestimated pain intensity despite increased analgesia administration. Furthermore, nurse attitudes, particularly concerns that analgesia might mask clinical deterioration significantly influenced treatment decisions, while patient factors including injury site, trauma multiplicity, and explicit analgesia requests independently predicted satisfaction. These findings collectively highlight the complex interplay between physiological severity, clinician perception, and patient expectations in achieving effective pain control.

Our results revealed a strong correlation between the physiological deterioration score MEWS and analgesic treatment. As the MEWS increased, the rate of analgesic treatment increased. Patients with MEWS ≤ 2 generally had milder injuries and typically reported lower pain levels, potentially reducing the perceived need for analgesia. However, those with MEWS > 2 had significantly greater pain levels. The stratification by MEWS revealed a complex pattern in pain management quality across severity levels. In the MEWS 3–4 group, the low analgesic treatment rate was due mainly to nurses’ underestimation of patients’ actual pain, mainly because nurses worried that analgesia might mask the true condition. This finding is consistent with previous research indicating that medical staff’s concerns about masking symptoms often lead to insufficient pain management in emergency settings (11). Notably, the most severe pain-perception gaps occurred in the MEWS 5–6 group, where despite substantially increased analgesia administration (44%), satisfaction remained low (34%) and patients reported significantly higher pain than nurses anticipated, suggesting that either analgesic interventions were inadequate or other factors diminished satisfaction. When MEWS ≥ 7, the analgesic effect improved significantly, and patients’ pain perception was lower than nurses’ expectations. Interestingly, the reverse perception gap in the MEWS ≥9 group, where nurses expected more pain than patients reported, coupled with relatively low satisfaction despite high analgesia rates, may indicate that physiological stabilization does not automatically ensure satisfaction, and highlights the need to address expectations and non-physical aspects of care in severely injured patients. This emphasizes the importance of accurate injury severity assessment for appropriate analgesic measures.

A survey of nurses’ attitudes toward pain control revealed that although they generally understand pain control, their actions are affected by concerns. The most prominent one is that analgesia may mask the underlying condition, which makes them reluctant to provide sufficient analgesia, especially for patients with moderate–severe injuries (MEWS 3–6). They also believe that pain may not cause additional adverse effects and consider it a non-top-priority issue. In contrast to some studies suggesting that nurses have a high level of pain control knowledge and positive attitudes (16–18), our research shows that there are still practical barriers to their implementation. For example, in the MEWS 3–4 group, the underestimation of pain and low analgesic treatment rate are manifestations of this attitude. To improve this, enhancing nurses’ training on pain assessment and the importance of pain control is essential.

Our attitude profile—high agreement that “analgesia may mask the clinical condition,” moderate endorsement that pain is not top-priority, and low skepticism toward patients—parallels international emergency room evidence. A mixed-studies review synthesizing emergency room evidence. Staff views identifies fear of masking diagnosis, diagnostic uncertainty, crowding, and “opiophobia” as recurring barriers to timely analgesia, cohering with our highest item score and the middling priority given to pain (19). Multicenter nurse surveys further show knowledge/attitude deficits under resource constraints and absent protocols, which can shift attention toward stabilizing physiology and away from systematic analgesia, consistent with our moderate rating for “not a top priority” and broad rejection of “trauma patients do not need pain control” (20).

Patient-related factors also significantly influence pain control. Females achieved better pain control than males did, possibly due to differences in pain tolerance and expression. This finding aligns with the results of previous studies that often report gender-based differences in pain perception and treatment (21). Patients who actively requested analgesia were more likely to have their pain controlled, highlighting the importance of patients’ self-reports. Multiple trauma patients received more pain control treatments, and the main injury site affected the pain control rate, with head and spine injuries having the highest rates, whereas limb injuries had the lowest rates. This is because injuries to vital parts attract more attention from nurses, and thus, more pain control measures are implemented. The results of the multiple regression analysis emphasized the importance of certain factors. The main injury site, injury type, analgesia request, and injury manifestation were all significant factors affecting the outcome.

Our linear regression showed higher satisfaction with an analgesia request and with obvious injury manifestations, but lower satisfaction for limb injuries. These patterns agree with ED data linking patient desire/receipt of analgesia to better pain relief and higher satisfaction—supporting the positive coefficient for “requested analgesia” (22). Visible injuries likely reduce diagnostic uncertainty and enable protocolized treatment, aligning with staff-reported enablers of pain care and explaining higher satisfaction when injury signs are obvious (19). Conversely, limb injuries (often long-bone fractures) show variable analgesic delivery; a recent national analysis reported only ~65% received any ED analgesic, suggesting care gaps that plausibly depress satisfaction, mirroring our negative coefficient for limb site (23).

Our findings are consistent with international and regional literature. A recent mixed-studies review from the UK synthesized staff perspectives and highlighted emergency room crowding, workflow pressure, competing priorities, diagnostic uncertainty, and “opiophobia” as persistent barriers to timely analgesia (19). In Australia, qualitative work likewise identified lack of time/resources, organizational constraints, and guideline–workflow misfit that deprioritize pain amid acute presentations (24). Implementation studies show that protocolized, nurse-initiated pathways raise analgesic administration yet overall rates remain low, underscoring structural bottlenecks (documentation gaps, reassessment lapses) despite education and protocols (25). Beyond systems, cultural factors shape equity: a US meta-analysis found racial/ethnic minorities less likely to receive emergency room analgesia for acute pain, pointing to implicit bias and language barriers that may also operate regionally (26, 27). Together, these data support our results that trauma-pain control is undermined by resource limitations (crowding, staffing, flow) and cultural/attitudinal concerns (fear of masking diagnosis, opioid stigma), and motivate context-adapted, protocolized, and equity-focused interventions in emergency room.

### 4.1 Limitation

This study has several limitations. The use of convenience sampling, while practical in the emergency setting, may introduce selection bias and limit the generalizability of our findings. The research was conducted at a single medical institution, so the sample may not fully represent the general situation of hospitals in different regions and of different levels, which may further limit the extrapolation of the research results. The research time was relatively short, making it difficult to comprehensively track the dynamic changes in pain control and related influencing factors during the entire treatment cycle of patients. Additionally, while MEWS provides valuable physiological data, it is not a pain-specific metric, and its relationship with pain experiences requires careful interpretation. Furthermore, this study focused mainly on nurses’ attitudes and behaviors toward pain control and explored less the roles of other medical team members, such as doctors, in pain management, failing to fully present the impact of multidisciplinary collaboration on pain control.

## 5 Conclusion

This study demonstrates that effective pain management in emergency trauma care requires addressing the complex interplay between physiological severity, clinicians’ perceptions and attitudes, and patient-specific factors. Rather than simply repeating our findings, we propose several forward-looking recommendations for emergency care policy and practice: First, institutional protocols should mandate structured pain assessment using validated tools for all trauma patients, regardless of physiological stability scores. Second, emergency departments should implement targeted training programs that address specific identified barriers, particularly nurses’ concerns about masking symptoms and underestimation of moderate pain. Third, clinical guidelines should emphasize a patient-centered approach that considers injury characteristics, explicit patient requests, and visible trauma manifestations when making analgesic decisions. Finally, we recommend integrating pain management quality indicators into emergency department performance metrics to prioritize institutional accountability. Future implementation research should evaluate the impact of these specific interventions on both clinical outcomes and patient experiences in emergency trauma care.

## Data Availability

The raw data supporting the conclusions of this article will be made available by the authors, without undue reservation.
